# SARS-CoV-2 Variants in Immunocompromised Patient Given Antibody Monotherapy

**DOI:** 10.3201/eid2710.211509

**Published:** 2021-10

**Authors:** Aurélie Truffot, Julien Andréani, Marion Le Maréchal, Alban Caporossi, Olivier Epaulard, Raphaele Germi, Pascal Poignard, Sylvie Larrat

**Affiliations:** Centre Hospitalier Universitaire Grenoble Alpes, Grenoble, France

**Keywords:** coronavirus disease, COVID-19, severe acute respiratory syndrome coronavirus 2, SARS-CoV-2, coronaviruses, viruses, respiratory infections, immunotherapy, bamlanivimab, monoclonal antibody, monotherapy, whole-genome sequencing, compartmentalization, selection, variants, zoonoses

## Abstract

A 72-year-old immunocompromised man infected with severe acute respiratory syndrome coronavirus 2 received bamlanivimab monotherapy. Viral evolution was monitored in nasopharyngeal and blood samples by melting curve analysis of single-nucleotide polymorphisms and whole-genome sequencing. Rapid emergence of spike receptor binding domain mutations was found, associated with a compartmentalization of viral populations.

A 72-year-old immunocompromised man in France who had chronic lymphocytic leukemia associated with hypogammaglobinemia for 4 years experienced diarrhea, asthenia, fever, and cough associated with coronavirus disease (COVID-19). Although he had received 1 injection of severe acute respiratory syndrome coronavirus 2 (SARS-CoV-2) mRNA vaccine (BNT162b2; Pfizer/BioNTech, https://www.pfizer.com) 20 days earlier, we confirmed a diagnosis of COVID-19 by using a semiquantitative SARS-CoV-2 reverse transcription PCR (RT-PCR) viral load assay. This assay showed a cycle threshold (C_t_) value of 27 for a nasopharyngeal swab specimen. His most recent monoclonal antibody (mAb) chemotherapy treatment (venetoclax and rituximab) had been conducted 17 days earlier. Because of his immunocompromised status, treatment with bamlanivimab (LY-CoV555), a neutralizing IgG1 mAb, was initiated at day 0, 4 days after onset of symptoms (Table). The patient received an infusion of 700 mg in a single dose and was discharged.

**Table:** Clinical and biological characteristics of immunocompromised patient given bamlanivimab for COVID-19, France*

Disease course, day†	Clinical manifestations	Treatment/action	Clinical samples‡	RT-PCR results (mean C_t_ value)	VirSNIP Kit results	NGS clade
‒20		First dose mRNA vaccine§				
‒17		Venetoclax, rituximab				
‒4	Cough, fever, diarrhea, asthenia	NA				
‒3			NP	Positive (27)¶	NA	NA
0		Bamlanivimab (700 mg)				
3			NP	Positive (20)	E484, N501Y	20I/501Y.V1
			Blood	Positive (37)	NA	NA
			Serum (30.7)			
6		Hospitalized at infectious diseases department	NP	Positive (21)	E484Q, N501Y	20I/501Y.V1 + E484Q
			Blood	Negative	NA	NA
7			Serum (23.2)			
10		Convalescent-phase plasma	NP	Positive (17)	E484Q, N501Y	20I/501Y.V1 + E484Q
11			NP	Positive (19)	E484Q, N501Y	20I/501Y.V1 + E484Q
			Blood	Positive (30)	E484, N501Y	20I/501Y.V1 ± 493R
		High-flow nasal oxygen	Serum (26.5)			
13		Transferred to ICU				
15			NP	Positive (21)	E484Q, N501Y	20I/501Y.V1+E484Q
17			Blood	Positive (31)	E484, N501Y	20I/501Y.V1 ± 493R ± 484K ± 484Q
			Serum (22.9)			
21		High-dose corticotherapy protocol				
26		High-dose corticotherapy protocol				
33		Transferred to infectious disease department	NP	Positive (17)	E484Q, N501Y	20I/501Y.V1 + E484Q
	Improvement in respiratory condition	NA	Blood	Positive (37)	NA	NA
			Serum (30.8)			
39			NP	Positive (17)	E484Q, N501Y	20I/501Y.V1 + E484Q
			Blood	Negative		
			Serum (18.6)			
45			NP	Positive (20)	E484Q, N501Y	20I/501Y.V1 + E484Q
47		Treatment with remdesivir (10 d)				
52			NP	Positive (31)	E484Q, N501Y	20I/501Y.V1 + E484Q
54			NP	Positive (30)	E484Q, N501Y	20I/501Y.V1 + E484Q
56		Hospitalization for follow-up care				
61			NP	Negative	NA	NA
80			NP	Negative	NA	NA

*Blank cells indicate that clinical status was stable on that day, and no treatment was given. COVID-19, coronavirus disease; Ct, cycle threshold; D, day; ICU, intensive care unit; NA, not available; NP, nasopharyngeal swab specimen; NGS, next-generation sequencing; RT-PCR, reverse transcription PCR.

†Day 0 indicates first day of follow-up care at hospital.

‡Serologic results given by using the Wantai antibody test (index of positivity = 1).

§Vaccine BNT162b2 (Pfizer/BioNTech, https://www.pfizer.com).

¶Test was performed in an external laboratory (no sample was available for further analysis).

Analysis of samples showed a high viral load in a nasopharyngeal swab specimen (C_t_ 20) and a blood sample (C_t_ 37) (Table). Three days after the mAb infusion, the patient’s symptoms worsened, and he was hospitalized in the Infectious Diseases Department at Grenoble Hospital (Grenoble, France) on day 6. The condition of the patient had deteriorated; he had an additional need for oxygen, which resulted in a convalescent-phase plasma transfusion on day 10. 

After this treatment, the condition of the patient continued to deteriorate, and he was transferred to the intensive care unit on day 13. A high dose of corticosteroids was given on days 21‒26. This treatment resulted in an improvement of his respiratory condition, but the patient remained dependent on supplemental oxygen (6 L/min). The patient was discharged from the intensive care unit and returned to the infectious disease department on day 33, but still had a high viral load in nasopharyngeal swab specimens (C_t_ v20 on day 45). 

Because of this persistent viral replication, the patient was given remdesivir on day 47 and this treatment was continued for 10 days (200 mg for 1 day, followed by 100 mg/d for 9 days). SARS-CoV-2 carriage in a nasopharyngeal swab specimen decreased during treatment, and the patient was discharged from the infectious disease department and transferred to a rehabilitation center. The nasopharyngeal swab specimen viral load became negative on day 61.

To monitor viral evolution, we performed a multiplex RT-PCR based on melting curve analyses with VirSNIP Kits (TIB Molbiol, https://www.tib-molbiol.de) to evaluate the presence of the S: E484K and S: N501Y mutations in SARS-CoV-2 variants. Three days after mAb treatment (day 3), RT-PCR results suggested the presence of S: N501Y and an absence of S:E484K on an nasopharyngeal swab specimen. On day 6, the S: N501Y mutation was still present but was also found associated with an undetermined mutation at position 484 (melting temperatures different from those of wild-type E and the mutated strain K). On day 11, we detected the S: N501Y mutation in a blood sample but found no mutation at position 484. No nasopharyngeal swab specimen or blood sample from before mAb administration was available for analysis and comparison.

We performed whole-genome sequencing on 12 clinical samples by using amplicon-based technology on the Ion Torrent Platform (ThermoFisher, https://www.thermofisher.com) according to the protocol of and plug-ins used by Sjaarda et al. (*1*). We confirmed results of this analysis by using the minimap2 program (*2*). This analysis detected clade 20I/501Y.V1, Alpha variant (Pangolin: B.1.1.7), on day 3 in nasopharyngeal swab specimens. Three days later (day 6), a novel mutation (G23012C, S: E484Q) appeared in nasopharyngeal swab specimens at frequency of 82%, which rapidly reached >99% (S: E484Q) 10 days after mAb treatment (Table; Figure). Eleven days after the mAb infusion, we detected an additional nucleotide mutation A23040G (S: Q493R) in only a blood sample at a frequency of 64%. This rate reached 76% at day 17 without any detection in nasopharyngeal swab specimens.

**Figure:**
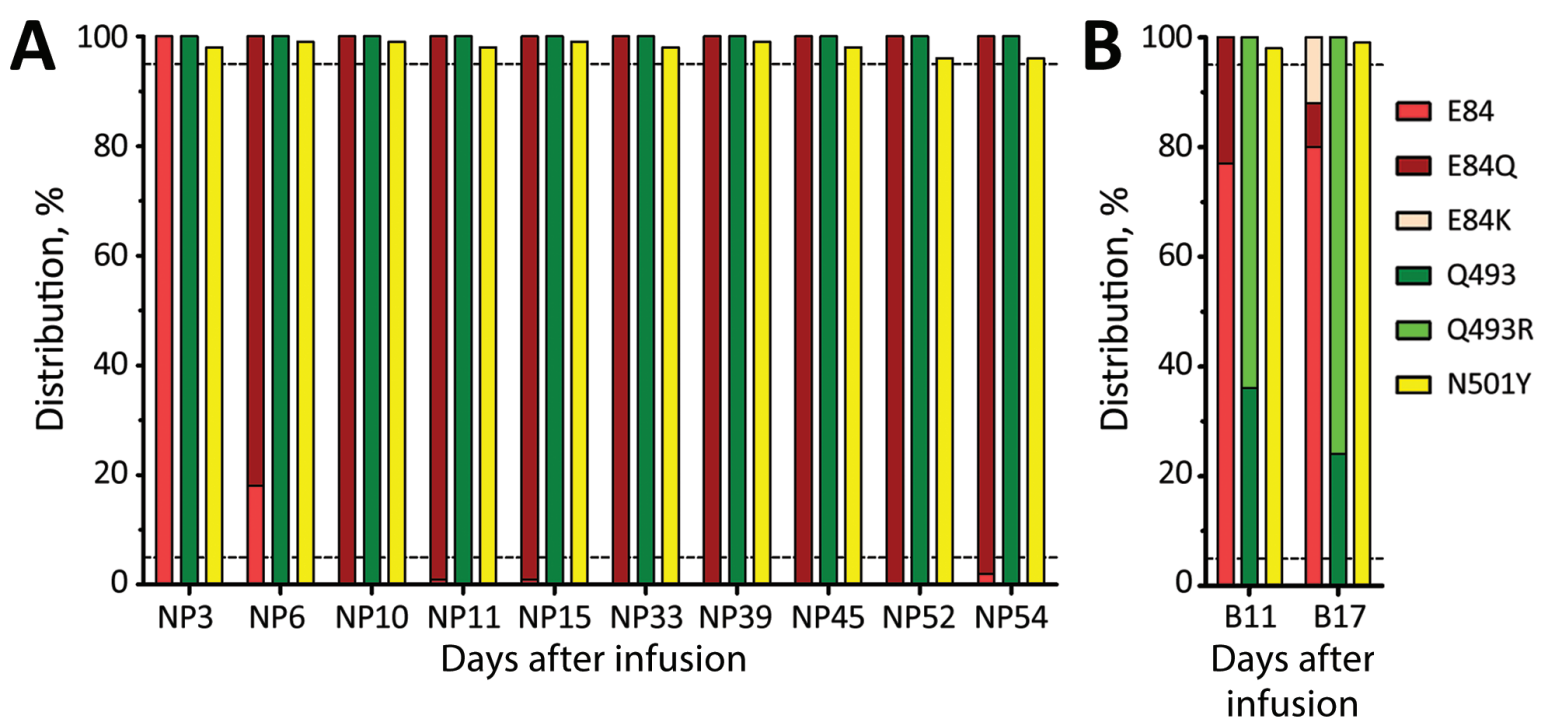
Severe acute respiratory syndrome coronavirus 2 variants in immunocompromised patient in France given antibody monotherapy showing compartmentalization and variation of mutation frequency for the spike protein. Mutations of interest are indicated by days after bamlanivimab infusion. A) NP samples; B) blood samples. The upper dashed horizontal line indicates 95% and the lower dashed horizontal line indicates 5%. B, blood; NP, nasopharyngeal.

Clinical trials of monotherapy treatment for SARS-CoV-2 infection have shown that subsequent dynamic shifts in the viral population appear to be frequent (*3*,*4*). An in vitro model showed that E484 and Q493 are 2 amino acid mutations of the spike protein that are known to be critical for bamlanivimab binding (*5*,*6*). The S: E484Q mutation is a hotspot of escape and could reduce susceptibility to bamlanivimab by >1,000-fold (*6*) and S: Q493R by >6,666-fold (*7*). Use of bitherapy with bamlanivimab and etesevimab decreases the risk for emergence of drug-resistant variants (*5*,*8*). However, an escape mutation after use of this drug combination was recently described (*7*).

Our analysis identified signs of compartmentalized viral populations on the basis of sequences recovered in blood and nasopharyngeal swab samples (notably on day 17). Such a phenomenon has been reported in clinical trials (*9*,*10*). Further analysis is needed to distinguish genetic changes that occur in the primary viral population from apparent changes to clarify whether such escape mutants are enough to spread and persist in humans and how SARS-CoV-2 displays compartmentalized replication. Genomic surveillance for SARS-CoV-2 variants is encouraged for COVID-19 patients given mAbs as monotherapy or biotherapy.
